# The Effect and Mechanism of Cholesterol and Vitamin B_12_ on Multi-Domain Cognitive Function: A Prospective Study on Chinese Middle-Aged and Older Adults

**DOI:** 10.3389/fnagi.2021.707958

**Published:** 2021-08-27

**Authors:** Lijing Wang, Kuo Liu, Xiaona Zhang, Yushan Wang, Wen Liu, Tao Wang, Ling Hao, Mengwei Ju, Rong Xiao

**Affiliations:** School of Public Health, Capital Medical University, Beijing, China

**Keywords:** mild cognitive impairment, cholesterol, vitamin B12, dose-response relation, mediation analysis

## Abstract

**Background:** Nutrients are associated with cognitive function, but limited research studies have systematically evaluated on multi-domain cognitive function. The aim of this study was to investigate the effect and mechanism of specific nutrient on multi-domain cognitive function, and provide nutrition guidance for improving cognitive function.

**Methods:** Participants were selected based on a multicenter prospective study on middle-aged and older adults in China. Global cognitive function was evaluated by the Mini-Mental State Examination (MMSE). Nutrients intake was assessed according to food frequency questionnaire and China Food Composition Database, and principal component analysis was performed to extract nutrient patterns. Associations between specific nutrients and cognitive function were assessed using log-binomial regression. Restricted cubic spline was used to illustrate the dose-response relationship of nutrients with multi-domain cognitive function. Mediation analysis was used to determine the mechanism of nutrients in cognitive function.

**Results:** Four nutrient patterns were identified (vitamin-mineral, protein-carbohydrate, fatty acid-vitamin E, and cholesterol-vitamin B_12_), and only a nutrient pattern rich in cholesterol and vitamin B_12_ was found associated with cognitive function (RR = 0.891, 95%CI = 0.794–0.999). In multi-domain cognitive function, dietary cholesterol and vitamin B_12_ were related to better performance of visual memory function (*P* = 0.034, *P* = 0.02). In dose-response relationship, it suggested a U-shaped association between vitamin B_12_ and MMSE (*P* = 0.02) within a certain range.

**Conclusions:** Dietary intake rich in cholesterol and vitamin B_12_ was associated with better cognitive function, and vitamin B_12_ had a U-shaped dose-response relation with MMSE. Thus, ensuring moderate cholesterol and vitamin B_12_intake may be an advisable strategy to improve cognitive function in middle-aged and older adults.

**Clinical Trial Registration:** EMCOA, ChiCTR-OOC-17011882, Registered 5th, July 2017-Retrospectively registered, http://www.medresman.org/uc/project/projectedit.aspx?proj=2610

## Introduction

Alzheimer's disease is one of the most common neurodegenerative diseases, characterized by progressive impairment in cognitive function, learning ability, memory function, and executive reasoning (Cui et al., 2020). Mild cognitive impairment (MCI) is a transitional stage between normal aging and dementia, 60–100% can develop into Alzheimer's disease (AD) within 5–10 years (Xue et al., 2018). According to the latest research, the overall prevalence of MCI and AD was estimated to be 15.5 and 3.9%, respectively, which imposed a considerable burden on individuals and society in China (Jia et al., 2020). Thus, early intervention on cognitive function can slow down the progress of AD efficiently (Fiorini et al., 2020).

In the past two or three decades, dietary habits in China have been experiencing a great change, particularly consumption of meat and sodium (Zhou et al., 2019). For example, residents in Beijing experienced a transition from traditional grain-based to animal-sourced diet (Xiong et al., 2019). Meantime, dietary habit transition leads to change in nutrient intake. Various studies have verified a strong link between nutrients and cognitive function, and most studies only focused on a single nutrient, while a single nutrient was not sufficient to affect cognitive function (Shi et al., 2019). Nutrient pattern may be more appropriate to examine the cumulative beneficial effect and common mechanism of nutrients in cognitive function, and it could explore the bioactive components of foods and potential interactions (Scarmeas et al., 2018). In Sweden, recent studies have found that nutrient pattern may play important roles in brain heath, but it still lacked relevant research in Chinese populations (Prinelli et al., 2019). Besides, the effects of nutrients on cognitive function may vary with intake doses, which revealed potential non-linear relationships between nutrients and cognitive function (Chianese et al., 2017). A dose-response meta-analysis showed a significant non-linear relationship between polyunsaturated fatty acids and MCI (Zhang et al., 2016). A prospective cohort study also found a non-linear relationship between dietary Mg intake and cognitive function in adult women aged 65–79 in the United States (Lo et al., 2019). Therefore, it is necessary to focus on the non-linear relationship between nutrients and cognitive function to provide a precise dietary recommendation.

Nowadays, increasing studies elucidated the mechanisms of nutrients affecting cognitive function, such as oxidative stress, inflammatory processes, and Aβ or tau protein accumulation (Poulose et al., 2017). Recently, cholesterol and its oxygenated derivatives were found to play an important role in cognitive impairment, such as 27-hydroxycholesterol (27-OHC) and 24S-hydroxycholesterol (24S-OHC) (Wang et al., 2016a). An animal research study proved that high fat/cholesterol diet resulted in increasing level of tau phosphorylation and memory loss (Bhat and Thirumangalakudi, 2013). Because of their role in homocysteine metabolism, the mechanisms of vitamin B_12_ affecting cognitive function were briefly summarized as cerebrovascular effects, activation of tau kinases, or inhibition of methylation reactions (Smith and Refsum, 2016). It showed that both cholesterol and vitamin B_12_ could affect cognitive function through tau phosphorylation; however, the findings are inconsistent and fragmented (Vauzour et al., 2017). Therefore, it is necessary to perform mediation analysis to verify the role of cholesterol oxygenated derivatives or tau phosphorylation between nutrients and cognitive function, and to provide an important basis for exploring the common mechanism between nutrients and cognitive function.

This study was performed mainly to demonstrate the dose-response relationship between specific nutrient in nutrient patterns and multi-domain cognitive function based on a multicenter prospective study, and explore the potential mechanisms, which could provide scientific dietary recommendations for middle-aged and older Chinese populations.

## Methods

### Participants

**Figure 1:**
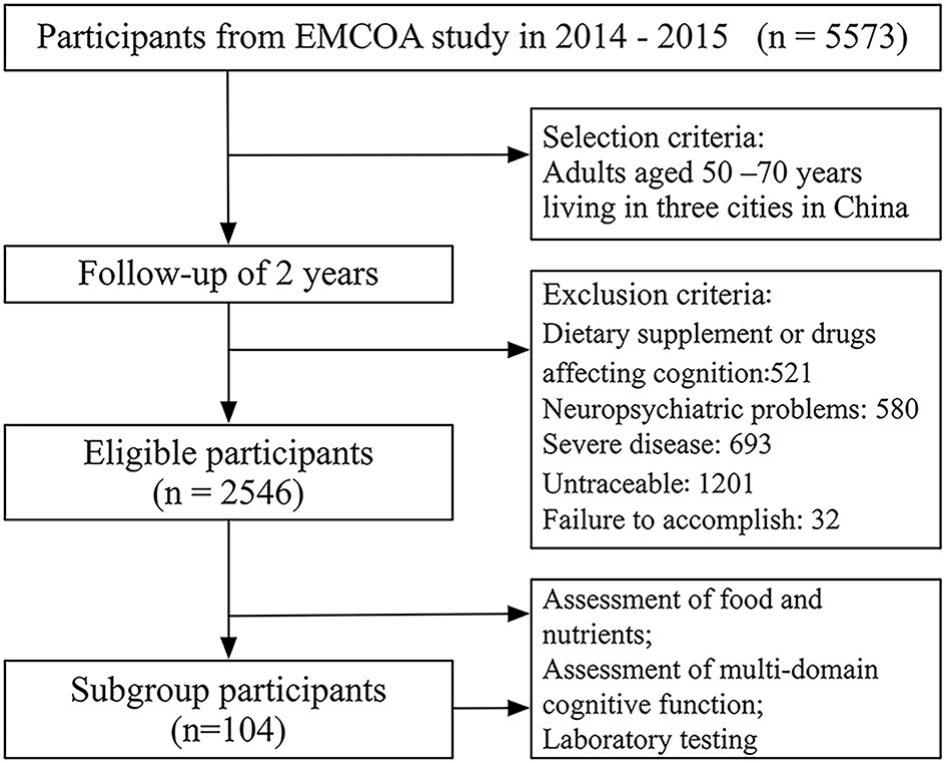
Flow diagram.

The Effects and Mechanism Investigation of Cholesterol and Oxysterol on Alzheimer's disease (EMCOA) study was a multicenter prospective study on community-dwelling volunteers initiated by Capital Medical University in 2014–2015 (Yu et al., 2018). In this study, participants aged 50 and 70 years were recruited and followed for an average of 2 years. Participants who had neuropsychiatric problems (e.g., depression, schizophrenia, drug addiction or other), severe diseases (e.g., visual impairment, hearing loss, or other severe organ dysfunction), who used dietary supplements or drugs affecting cognitive function, who were untraceable or failure to accomplish were excluded. Finally, 2,546 middle-aged and older eligible adults were selected for the demonstration of the relationship between specific nutrients in nutrient pattern, food composition, and multi-domain cognitive function. A subgroup that included 104 participants was randomly selected to explore the mechanism of nutrients in cognitive function. The details are shown in Figure 1. Face-to-face interviews were conducted by a survey team composed of clinical neuropsychologists and research investigators who have been trained in the details of the measurements and questionnaires. The assessments of diet and cognitive function was conducted initially, and then after 2 years. The EpiData software was used for parallel double data entry.

### Assessment of Food Composition and Nutrients

Dietary intake was assessed with a semiquantitative Food Frequency Questionnaire (FFQ) (Clare et al., 2017), which included 85 different foods types of 33 items. The participants were requested to state the frequency (per year, month, week, or day) and amount (in grams, bowls, etc.) of food intake for each food item on baseline, and follow up to make sure they were on the same food regimen during the study. For each food reported, food models and an album with over 50 photos of the most common dietary products were used as helpful tools to determine the amounts of food portions. Nutrient intake was calculated by multiplying the nutrient content of each food of specific portion size by the frequency of consumption and then summed over all food items from the China Food Composition Database (CFCD) (Rong et al., 2017). In this study, the following nutrient intake was calculated: protein, carbohydrate, dietary fiber, cholesterol, saturated fatty acid (SFA), monounsaturated fatty acid (MUFA), polyunsaturated fatty acid (PUFA), vitamin A, vitamin B_1_, vitamin B_2_, niacin, vitamin B_6_, folic acid, vitamin B_12_, vitamin C, vitamin E, magnesium, iron, zinc, selenium, copper, and manganese. The food composition included meat, milk and its products, eggs, grain, fruits, vegetables, etc.

### Assessment of Cognitive Function

The global cognitive function of the participants was assessed by Mini-Mental State Examination (MMSE). MMSE is commonly used as a simple tool to screen for cognitive impairment based on education level. The diagnostic criteria used for MCI screening were as follows: (1) illiterate individuals ≤ 19 points, (2) individuals with 6 or less years of education ≤ 22 points, (3) individuals with 7 or more years of education ≤ 26 points. Then, if the participants were suspected of having MCI based on their MMSE performance, they would be comprehensively examined by neurologists to establish a clinical diagnosis (Wong et al., 2016; Yin et al., 2018; Jiang et al., 2020).

Comprehensive neuropsychological measures were used to assess the multi-domain cognitive function (Yu et al., 2018). Verbal memory function was assessed by Auditory Verbal Learning Test (AVLT) using summarized scores of immediate recall (AVLT-IR), short recall (AVLT-SR), and long recall (AVLT-LR) (Hong et al., 2015); attention/processing speed/executive function was assessed by Logical Memory Test—immediate recall (LMT-IR) (Wang et al., 2016b), Digit Span Forward (DSTF), and Digit Span Backward (DSTB) (Darby et al., 2017), and Trail Making Tests (TMT) A and B (Wei et al., 2018) from Wechsler Memory Scale Revised for China (WMS-RC); flexibility of cognitive function was assessed by the Stroop Color-Word Test-Interference Trial (SCWT-IT) (Bondi et al., 2002); and visual memory function was assessed by the Picture Memories Test (PMT) from WMS-RC (Krakowski and Czobor, 2011). The measurements were performed in a quiet and private room by trained technicians.

### Covariates

The questionnaires on sociodemographic included age, sex, education (in years), body mass index (BMI); lifestyle risk factors included smoking, drinking, and reading habit; and medical history included hypertension (measured blood pressure > 140/90 mmHg or antihypertensive medication), hyperlipidemia (total cholesterol ≥ 6.2 mmol/L or low-density lipoprotein ≥ 4.1 mmol/L or high-density lipoprotein < 1 mmol/L or triglycerides ≥ 2.3 mmol/L or lipid-lowering medication), diabetes (fasting glucose ≥ 7 mmol/L or antidiabetic medication), and cerebrovascular diseases (CVD). Smokers were defined as those who have smoked at least one cigarette per day in the last 6 months. Alcohol drinkers were defined as those who have consumed any type of alcoholic beverage at least once a week in the last 6 months. Weight and height were measured according to World Health Organization recommendations, and BMI was calculated as weight in kilograms divided by height in meters squared (kg/m^2^) (Physical Status, 1995).

### Laboratory Testing

After at least 12 h of fasting, blood samples were collected in 6 ml tubes containing coagulant and inverted 8–10 times. Serum was obtained by centrifugation at 3,000 rpm at 4°C for 10 min, and stored at −80°C until use. The serum concentrations of 27-OHC and 24S-OHC were measured by high performance liquid chromatography–mass spectrometry (HPLC-MS). A standard curve was generated and used to calculate the concentrations of 27-OHC and 24S-OHC with internal standard, according to the method previously described (Bandaru and Haughey, 2014). Serum total tau (t-tau) and phosphorylated tau (p-tau) were measured using a commercial enzyme-linked immunosorbent assay (ELISA) kit, and the concentration of the samples was determined by comparing the standard curve (Tang et al., 2019).

### Statistical Analysis

Statistical analyses were performed using STATA version 15.0 (Stata Corp, College Station, TX, United States), R statistical software version 3.6.1 (R Foundation, Vienna, Austria), and IBM SPSS Statistics 23.0 software (SPSS, Chicago, IL, United States). A two-sided *P* ≤ 0.05 was considered statistically significant.

Continuous variables were expressed as median (interquartile range) and compared by Mann–Whitney *U*-test. Categorical variables were presented as number (percentages) and compared by chi-square test or Fisher's exact test. Principal component analysis (PCA) was performed to identify nutrient patterns. Varimax rotation was used in component analysis, and eigenvalues > 1 were considered as principal component (Makura-Kankwende et al., 2020). Log-binomial regression was used to estimate the relationship between nutrient patterns and MCI (Palta et al., 2018). Multiple linear regression was used to illustrate the linear relationship between specific nutrients in nutrient patterns and multi-domain cognitive function. Restricted cubic spline (RCA) was used to flexibly model the dose-response relationship and explore the potential non-linear association between specific nutrients in nutrient patterns and multi-domain cognitive function (Desquilbet and Mariotti, 2010).

## Results

### Characteristics of Participants

General characteristics, nutrients intake, and multi-domain cognitive function were compared between participants with mild cognitive impairment (MCI) and those with normal cognition (NC). The details are shown in Supplementary Table 1. Of the 2,546 participants, 54.6% were women. It showed that the difference of age between the MCI and NC groups was significant, but the median was same (*P* = 0.016). Compared with NC, participants in the MCI group had a worse performance in multi-domain cognitive function (*P* < 0.001). No significant differences were observed in the other characteristics.

### Association Between Nutrient Patterns and MCI

Four independent nutrient patterns were identified by principal component analysis (PCA) (Table 1). The first nutrient pattern was identified as “Vitamin-Mineral,” explained 36.4% of the variance and was illustrated by intakes of vitamin A, vitamin B_2_, vitamin B_6_, folic acid, vitamin C, magnesium, iron, zinc, copper, and manganese. The second nutrient pattern was identified as “Protein-Carbohydrate,” explained 21.6% of the variance, and was illustrated by intakes of protein, carbohydrate, dietary fiber, and vitamin B_1_. The third nutrient pattern was identified as “Fatty Acid-Vitamin E,” explained 17.2% of the variance, and was illustrated by intakes of SFA, MUFA, PUFA, and vitamin E. The fourth nutrient pattern was identified as “Cholesterol-Vitamin B_12_,” explained 12.2% of the variance, and was illustrated by intakes of cholesterol and vitamin B_12_.

**Table 1:** Principal component analysis of nutrient patterns.

**Variables**	**Vitamin-mineral**	**Protein-carbohydrate**	**Fatty acid-vitamin E**	**Cholesterol-vitamin B_**12**_**
Protein	0.260	**0.702**	0.401	0.449
Carbohydrate	0.145	**0.945**	0.079	0.083
Dietary fiber	0.261	**0.841**	0.112	−0.027
Cholesterol	0.036	0.159	0.132	**0.842**
SFA	0.040	0.191	**0.735**	0.574
MUFA	0.051	0.068	**0.965**	0.016
PUFA	0.021	0.204	**0.816**	0.429
Vitamin A	**0.900**	0.146	−0.024	0.225
Vitamin B_1_	0.392	**0.810**	0.169	0.253
Vitamin B_2_	**0.864**	0.260	0.085	0.275
Niacin	0.600	0.442	0.480	0.206
Vitamin B_6_	**0.922**	0.210	0.072	0.063
Folic acid	**0.917**	0.317	0.151	0.068
Vitamin B_12_	0.121	0.013	0.066	**0.861**
Vitamin C	**0.944**	0.184	−0.017	−0.022
Vitamin E	0.259	0.185	**0.872**	−0.058
Magnesium	**0.671**	0.524	0.451	0.093
Iron	**0.903**	0.385	0.084	0.055
Zinc	**0.706**	0.556	0.283	0.284
Selenium	0.579	0.507	0.251	0.521
Copper	**0.763**	0.519	0.322	0.096
Manganese	**0.693**	**0.611**	0.304	0.033
Iodine	0.552	−0.055	0.070	−0.062

*Bold entries indicate measures with high loadings on each factor*.

*SFA, saturated fatty acid; MUFA, monounsaturated fatty acid; PUFA, polyunsaturated fatty acid*.

Log-binomial regression was used to determine the effects of the different nutrient patterns on mild cognitive impairment (MCI). The results showed that only the Cholesterol-Vitamin B_12_ nutrient pattern (RR = 0.891, 95%CI = 0.794–.999, *P* = 0.048) was negatively associated with MCI (Table 2).

**Table 2:** Log-binomial regression of nutrient patterns and MCI.

**Nutrient patterns**	**RR (95% CI)**	***P*-value**
**Unadjusted**
Vitamin-mineral	1.024 (0.933, 1.122)	0.622
Protein-carbohydrate	0.970 (0.877, 1.073)	0.557
Fatty acid-vitamin E	0.994 (0.901, 1.097)	0.907
Cholesterol-vitamin B_12_	0.892 (0.796, 1.000)	0.051
**Model 1**
Vitamin-mineral	1.016 (0.926, 1.115)	0.734
Protein-carbohydrate	0.967 (0.873, 1.072)	0.527
Fatty acid-vitamin E	0.993 (0.900, 1.095)	0.885
Cholesterol-vitamin B_12_	0.894 (0.797, 1.003)	0.055
**Model 2**
Vitamin-mineral	1.021 (0.931, 1.119)	0.665
Protein-carbohydrate	0.963 (0.868, 1.068)	0.477
Fatty acid-vitamin E	0.994 (0.902, 1.096)	0.906
Cholesterol-vitamin B_12_	0.891 (0.794, 0.999)	0.048^*^

*Model 1 was adjusted for age, sex, education, BMI, smoking, and drinking; Model 2 was adjusted for Model 1 and hypertension, hyperlipidemia, diabetes, and CVD*.

*MCI, mild cognitive impairment; BMI, body mass index; CVD, cerebrovascular diseases; RR, risk ratio; CI, confidence interval*.

* P <0.05, ^**^P < 0.001.

### Association Between Cholesterol or Vitamin B_12_ and Multi-Domain Cognitive Function

Multiple linear regression models were used to further examine the association between baseline cholesterol or vitamin B_12_ and follow-up multi-domain cognitive function. The models were adjusted for age, sex, education, BMI, smoking, drinking, hypertension, hyperlipidemia, diabetes, CVD, and other nutrient patterns (Table 3).They showed that cholesterol was positively associated with PMT (β = 0.137; *P* = 0.034), and that vitamin B_12_ was also related to better performance in PMT (β = 0.08; *P* = 0.02).

**Table 3:** Linear relationship between cholesterol or vitamin B_12_ and multi-domain cognitive function.

**Variables**	**Cholesterol**		**Vitamin B**_****12****_	
	**β (95% CI)**	***P*-value**	**β (95% CI)**	***P*-value**
MMSE	0.024 (−0.053, 0.100)	0.547	0.023 (−0.018, 0.064)	0.269
AVLT-IR	0.024 (−0.153, 0.200)	0.792	0.027 (−0.067, 0.122)	0.567
AVLT-SR	0.072 (−0.020, 0.164)	0.125	0.037 (−0.012, 0.086)	0.139
AVLT-LR	0.005 (−0.098, 0.108)	0.930	0.003 (−0.058, 0.052)	0.915
SDMT	0.154 (−0.253, 0.560)	0.459	−0.012 (−0.229, 0.205)	0.913
LMT	−0.029 (−0.234, 0.177)	0.784	0.034 (−0.076, 0.143)	0.544
TMTA	0.018 (−0.898, 0.934)	0.970	0.045 (−0.444, 0.535)	0.856
TMTB	−0.640 (−3.255, 1.975)	0.631	−0.326 (−1.723, 1.071)	0.647
DSTF	−0.010 (−0.061, 0.041)	0.697	0.013 (−0.014, 0.040)	0.353
DSTB	−0.027 (−0.074, 0.020)	0.260	−0.019 (−0.044, 0.007)	0.149
PMT	0.137 (0.010, 0.263)	0.034^*^	0.080 (0.013, 0.148)	0.020^*^
SCWT-IT	0.447 (−0.284, 1.179)	0.230	0.042 (−0.348, 0.432)	0.832

*Adjusted for age, sex, education, BMI, smoking, drinking, hypertension, hyperlipidemia, diabetes, CVD, and other nutrient patterns using multiple linear regression models*.

*MMSE, Mini-Mental State Examination; AVLT-IR, Auditory Verbal Learning Test—immediate recall; AVLT-SR, Auditory Verbal Learning Test—short recall; AVLT-LR Auditory Verbal Learning Test—long recall; SDMT, Symbol Digit Modalities Test; LMT, Logical Memory Test; TMTA (B), Trail Making Test A (B); DSTF, digit span test forwards; DSTB, digit span test backwards; PMT, Picture Memories Test; SCWT-IT, Stroop Color-Word Test Interference Trial; BMI, body mass index; CVD, cerebrovascular diseases; CI, confidence interval*.

* P < 0.05. ^**^P < 0.001.

**Figure 2:**
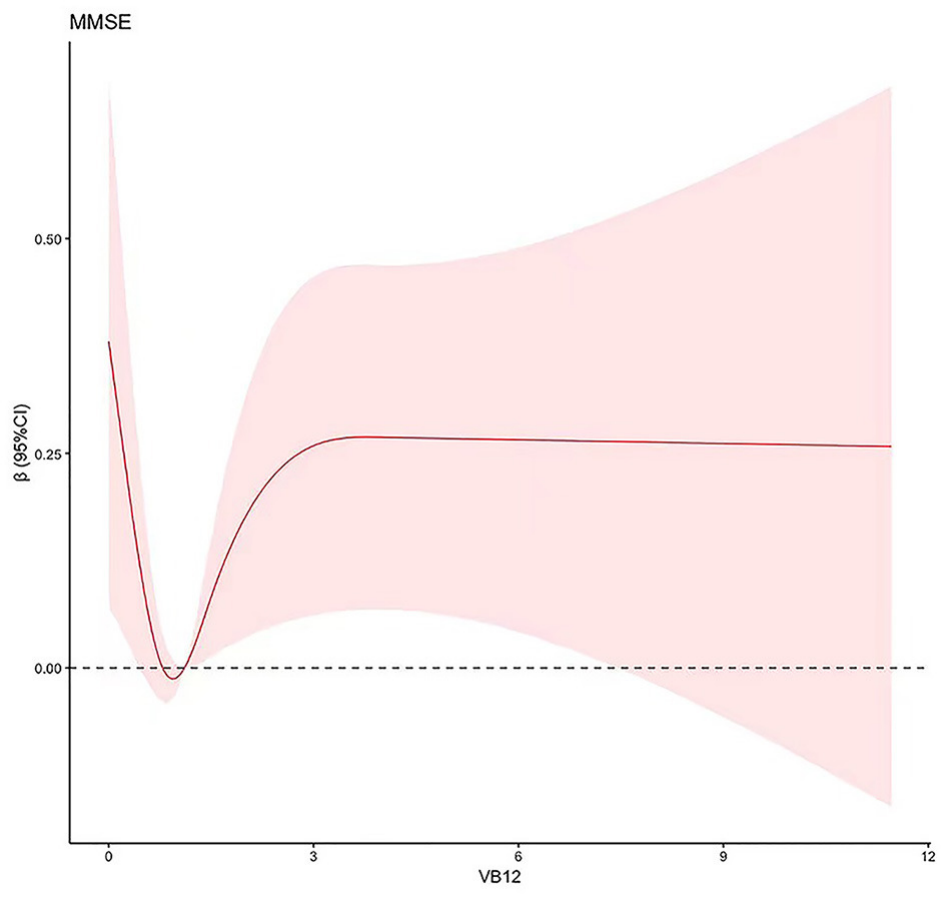
Dose-response relationship between vitamin B_12_ and MMSE. Adjusted for age, sex, education, BMI, smoking, drinking, hypertension, hyperlipidemia, diabetes, CVD, and other nutrient patterns using RCS models. The shaded area represents the estimated relative risk and the 95% CI. MMSE, Mini-Mental State Examination; BMI, body mass index; CVD, cerebrovascular diseases; CI, confidence interval.

Restricted cubic spline analysis was performed to simulate the non-linear dose-response relationship between cholesterol or vitamin B_12_ and multi-domain cognitive function. The models were adjusted for age, sex, education, BMI, smoking, drinking, hypertension, hyperlipidemia, diabetes, CVD, and other nutrient patterns (Table 4). The results showed that vitamin B_12_ had a non-linear relationship with MMSE (*P* = 0.020). Regarding the dose-response curve, it emerged a *U*-shaped relationship between vitamin B_12_ and MMSE when the intake was <3 μg/day (Figure 2). No dose-response relationship between cholesterol and multi-domain cognitive function was found.

**Table 4:** Dose-response relationship between cholesterol or vitamin B_12_ and multi-domain cognitive function.

**Variables**	**Cholesterol**		**Vitamin B**_****12****_	
	***P*-overall**	***P*-non-linear**	***P*-overall**	***P*-non-linear**
MMSE	0.221	0.111	0.036^*^	0.020^*^
AVLT-IR	0.172	0.092	0.144	0.107
AVLT-SR	0.204	0.313	0.587	0.966
AVLT-LR	0.482	0.295	0.894	0.767
SDMT	0.896	0.801	0.661	0.483
LMT	0.988	0.936	0.529	0.417
TMTA	0.633	0.435	0.713	0.563
TMTB	0.559	0.356	0.658	0.516
DSTF	0.957	0.990	0.096	0.067
DSTB	0.650	0.771	0.718	0.654
PMT	0.308	0.868	0.161	0.712
SCWT-IT	0.924	0.866	0.550	0.349

*Adjusted for age, sex, education, BMI, smoking, drinking, hypertension, hyperlipidemia, diabetes, CVD, and other nutrient patterns using restricted cubic spline (RCS) models*.

*MMSE, Mini-Mental State Examination; AVLT-IR, Auditory Verbal Learning Test—immediate recall; AVLT-SR, Auditory Verbal Learning Test—short recall; AVLT-LR Auditory Verbal Learning Test—long recall; SDMT, Symbol Digit Modalities Test; LMT, Logical Memory Test; TMTA (B), Trail Making Test A (B); DSTF, digit span test forward; DSTB, digit span test backward; PMT, Picture Memories Test; SCWT-IT, Stroop Color-Word Test Interference Trial; BMI, body mass index; CVD, cerebrovascular diseases; CI, confidence interval*.

* P < 0.05, ^**^P < 0.001.

### Mediation Effect Between Cholesterol or Vitamin B_12_ and Cognitive Function

A total of 104 participants were selected in the subgroup to elucidate how cholesterol and vitamin B_12_ affect cognitive function. The characteristics between overall samples used in previous analysis (n = 2546) and subgroup (n = 104) were demonstrated in Supplementary Table 2. It was showed that there was a significant difference in sex, BMI or drink habit between subgroup and overall, so these variants were adjusted in the analysis subsequently.

In mediation analysis, the average causal mediation effect (ACME) between cholesterol or vitamin B_12_ and MMSE was not significant when 27-OHC, 24S-OHC, t-tau, or p-tau was used as a mediator (*P* > 0.05). None of the mediators had significance in the relationship between nutrients and cognition. The details are shown in Supplementary Table 3.

## Discussions

In this prospective study based on middle-aged and older adults, we examined the effect of nutrient patterns on cognitive function. Four nutrient patterns were identified, and only the Cholesterol-Vitamin B_12_ pattern was associated with MCI (RR = 0.891, 95%CI = 0.794, 0.999). In multi-domain cognitive function, cholesterol and vitamin B_12_ were positively correlated with PMT (*P* = 0.034, *P* = 0.02). Furthermore, vitamin B_12_ was non-linearly associated with MMSE and the dose-response curve emerged U-shaped (*P* = 0.02). Generally, this study provided a perspective that nutrition, as a modifiable factor, could be an applicable and critical strategy to improve cognitive function.

In this study, a diet rich in cholesterol was associated with better cognitive function. Similarly, a study based on Framingham Heart Study showed that lower total cholesterol was correlated with poorer performance on cognitive function, and another cohort study showed that cholesterol was associated with deceased risk of dementia (Elias et al., 2005; Mielke et al., 2005). Moreover, cholesterol intake restriction had been removed from the 2015–2020 Dietary Guidelines in America, which indicates that the effect of cholesterol on cognitive function is complicated, and it was still difficult to make a conclusion whether it was harmful or beneficial (Williams et al., 2015). In multi-domain cognitive function, dietary cholesterol was found to be associated with PMT in this study. PMT was a subtest of WMS-RC, which was used to assess visual memory function. Little research has examined the relationship between dietary cholesterol and visual memory function, and a study has found that a mild increase in low-density lipoprotein cholesterol or high-density lipoprotein cholesterol level is related to a better visual memory (Kinno et al., 2019; Guo et al., 2020). Therefore, maintaining moderate cholesterol consumption may be advisable for cognitive function.

In this study, vitamin B_12_ was non-linearly associated with MMSE when the intake dose was within a certain range, which suggested that it was important to ensure adequate consumption of vitamin B_12_. A cross-sectional study in Ireland also proposed that dietary vitamin B_12_ was related with MMSE in older adults, indicating that vitamin B_12_ may be a protective factor for cognitive function (O'Connor et al., 2020). Meantime, cross-sectional data from a nationally representative study in the United States showed that low vitamin B_12_ was related to cognitive impairment independently among older adults (Molloy, 2020). Therefore, ensuring adequate vitamin B_12_ intake might be an effective intervention to maintain cognitive function. In addition, in this study, vitamin B_12_ was related to a better performance in PMT. Although no direct evidence between vitamin B_12_ and visual memory had been found, a community-based cross-sectional study demonstrated that elevated homocysteine was associated with poorer performance in visual memory test (Ai-Vyrn et al., 2008). More studies are needed to be conducted on the possible mechanisms between vitamin B_12_ and visual memory function.

In this study, the cholesterol-vitamin B_12_ nutrient pattern was associated with MCI, which led us to consider the common mechanism of cholesterol and vitamin B_12_ in cognitive function. However, no statistical evidence for the hypothesis that cholesterol and vitamin B_12_ could work together to affect cognitive function through cholesterol oxygenated derivatives or tau phosphorylation. First, mediation analysis can only identify mediators that had a linear mediation effect, but the biological process was complex, and the relationship might be non-linear. Next, cholesterol and vitamin B_12_ may affect cognitive function through other mechanisms. For instance, it has been revealed that both cholesterol and vitamin B_12_ could influence cognitive function *via* Aβ metabolism (Alam et al., 2017; Wang et al., 2019). What is more, an inflammatory effect played an important role in the process of dietary cholesterol or vitamin B_12_ affecting cognitive function (Chen et al., 2018; Ma et al., 2019). Additionally, increasing research studies examined the role of epigenetics in biological mechanism of AD. As an essential cofactor of homocysteine metabolism, vitamin B_12_ is integral to DNA methylation and consequently involved in the pathology of AD (Athanasopoulos et al., 2016). Recently, a cohort study in Scotland also found that cholesterol was associated with apolipoprotein E (APOE) methylation (Mur et al., 2020). Therefore, future studies may focus on exploring the common mechanism of cholesterol and vitamin B_12_ affecting cognitive function, especially in the role of epigenetics.

In this study, the nutrient pattern characterized as cholesterol and vitamin B_12_ was related to lower risk of MCI, and the possible biological explanation for this beneficial effect may come from the high loadings of cholesterol and vitamin B_12_. Mostly, dietary cholesterol and vitamin B_12_ are derived from meat, eggs, and milk. In this study, we explore the relationship of meat intake with cognitive function further, and it was found that meat intake was correlated with lower risk of MCI (Supplementary Table 4) and better performance of global cognitive function (Supplementary Table 5). Similarly, a longitudinal study on community dwellers in the state of New York also demonstrated that higher meat consumption was associated with better cognitive performance (Crichton et al., 2015). Meantime, a prospective study on oldest old Chinese population suggested that participants who consumed more meat were less likely to develop cognitive impairment (An et al., 2019). However, a cohort study from Sweden showed that adherence to a diet low in meat might contribute to healthy cognitive aging, the inconsistent results indicated that native conditions are needed to be considered in dietary instructions (Titova et al., 2013). The inconsistent evidence provides us a hypothesis that the effect of meat on cognitive function was complicated, and that it may be different regarding local conditions. In general, meat should be consumed appropriately, for it is a risk factor in other diseases such as cardiovascular disease.

This study has several strengths. First, the data in this study were based on a prospective study with a relatively large sample size. Second, cognitive function was assessed through multiple scales, such as verbal memory cognitive, attention/processing speed/executive function, flexibility cognitive function, and visual memory function, which would reflect cognitive function more comprehensively. Third, not only the linear effects of nutrients but also the non-linear effects on multi-domain cognitive function were evaluated. The limitations of this study are as follows: First, the number of APOE-ε4 alleles is a major non-modifiable risk factor for AD, but we did not measure the APOE genotype in this study. Recently, a national wide cross-sectional study in China observed that lifestyle such as dietary pattern was associated with cognitive function regardless of APOE genotype (Jin et al., 2021). In order to confirm the relationship between dietary intake and cognitive function, the research team will further explore it. Second, cognitive function was assessed by scales instead of clinical examination, and education level can significantly affect the performance in completing the scales. Thus, we adjusted education level in each statistical model to compensate for this limitation. Besides, the mediation effect of oxysterols or tau protein between cholesterol or vitamin B_12_ and MMSE was only examined by statistical analysis, but the biological process was complex, and the relationship might be non-linear. Therefore, animal experiments and *in vitro* studies are still needed to elucidate the molecular pathway and physiological regulation underlying the observed associations in the next step.

## Conclusions

In summary, the study provided evidence that a nutrient pattern rich in cholesterol and vitamin B_12_ was associated with lower risk of MCI and better performance in visual memory function. Vitamin B_12_ was non-linearly associated with MMSE, and the curve emerged *U*-shaped when the intake was within a certain range, which suggested that it was essential to establish a dose-response relationship for optimal dietary recommendations. Thus, ensuring appropriate cholesterol and vitamin B_12_ intake in the diet may be an effective strategy to prevent MCI in middle-aged and older adults. As a modifiable factor in cognitive function intervention, scientific dietary guidelines are critical.

## Data Availability Statement

The datasets analyzed during the current study are available from the corresponding author on reasonable request.

## Ethics Statement

The studies involving human participants were reviewed and approved by the Ethics Committee of Capital Medical University (2013SY35). The patients/participants provided their written informed consent to participate in this study. Written informed consent was obtained from the individual(s) for the publication of any potentially identifiable images or data included in this article.

## Author Contributions

RX conceived and designed the study. LW and KL performed statistical analysis and wrote the manuscript. XZ, YW, WL, TW, LH, and MJ helped in the collection of data and conducted the experiments. All authors contributed to the article and approved the submitted version.

## Conflict of Interest

The authors declare that the research was conducted in the absence of any commercial or financial relationships that could be construed as a potential conflict of interest.

## Publisher's Note

All claims expressed in this article are solely those of the authors and do not necessarily represent those of their affiliated organizations, or those of the publisher, the editors and the reviewers. Any product that may be evaluated in this article, or claim that may be made by its manufacturer, is not guaranteed or endorsed by the publisher.

## Abbreviations

27-OHC: 27-hydroxycholesterol
24S-OHC: 24S-hydroxycholesterol
ACME: average causal mediation effect
AD: Alzheimer's disease
Aβ: β-amyloid
AVLT-LR: auditory verbal learning test-long recall
AVLT-IR: auditory verbal learning test-immediate recall
AVLT-SR: auditory verbal learning test-short recall
BMI: body mass index
CFCD: China Food Composition Database
CVD: cerebrovascular diseases
DSTB: Digit span backwards
DSTF: Digit span forwards
EMCOA: effects and mechanism of cholesterol and oxysterol on Alzheimer's disease
FFQ: Food Frequency Questionnaire
LMT-IR: logical memory test-immediate recall
MCI: mild cognitive impairment
MMSE: Mini-Mental State Examination
MUFA: monounsaturated fatty acid
NC: normal cognition
PCA: principal component analysis
PMT: picture memories test
p-tau: phosphorylated tau
PUFA: polyunsaturated fatty acid
RCA: restricted cubic spline
SCWT-IT: Stroop Color-Word Test Interference Trial
SFA: saturated fatty acid
TMT: Trail Making Test
t-tau: total tau
WMS-RC: Wechsler Memory Scale-Revised of China.
