# Supplementary material for: The Effect and Mechanism of Cholesterol and Vitamin B12 on Multi-Domain Cognitive Function: A Prospective Study on Chinese Middle-Aged and Older Adults
Source: Front Aging Neurosci. 2021 Aug 27;13:707958. doi: 10.3389/fnagi.2021.707958 (PMC8430239; doi:10.3389/fnagi.2021.707958)
Supplement: Supplementary file 1 [file Table_1.DOCX]

Supplementary Material

# Supplementary Tables

**Supplementary Table 1 Comparison of characteristics between MCI and NC group**

| **Variables** | **MCI** | **NC** | ***P* value** | |
| --- | --- | --- | --- | --- |
| **General characteristics** | | | |  |
| Age | 60 (56,63) | 60 (57,64) | 0.016^*^ | |
| Sex  Male, *n* (%)  Female, *n* (%) | 143 (40.6%)  209 (59.4%) | 1000 (46.2%)  1164 (53.8%) | 0.057 | |
| Education years | 9 (9,12) | 9 (9,12) | 0.120 | |
| BMI (kg/m^2^) | 24.3 (22.6,26.1) | 24.5 (22.8,26.6) | 0.118 | |
| Smoking, *n* (%) | 82 (23.3%) | 555 (25.6%) | 0.622 | |
| Drinking, *n* (%) | 172 (48.9%) | 1008 (46.6%) | 0.461 | |
| Hypertension, *n* (%) | 119 (33.8%) | 785 (36.3%) | 0.626 | |
| Hyperlipidemia, *n* (%) | 84 (23.9%) | 646 (29.9%) | 0.061 | |
| Diabetes, *n* (%) | 63 (17.9%) | 388 (17.9%) | 1.000 | |
| CVD, *n* (%) | 15 (4.3%) | 50 (2.3%) | 0.097 | |
| **Nutrients intake** | | | |  |
| Cholesterol, mg | 268.5(153.8,320.5) | 276.8 (155.8,342.7) | 0.094 | |
| Vitamin B_12_, μg | 1.0 (0.6,1.6) | 1.1(0.6,1.8) | 0.087 | |
| **Multi-domain cognitive function** | | | |  |
| MMSE | 25 (24,26) | 29 (28,29) | < 0.001^**^ | |
| AVLT-IR | 13 (10,16) | 15 (12,18) | < 0.001^**^ | |
| AVLT-SR | 4 (3,6) | 5 (4,7) | < 0.001^**^ | |
| AVLT-LR | 3 (1,5) | 4 (3,6) | < 0.001^**^ | |
| SDMT | 32 (25,39) | 35 (28,44) | < 0.001^**^ | |
| LMT | 7 (3.5,10.5) | 11 (7,15) | < 0.001^**^ | |
| TMTA | 70.5 (57.0,92.3) | 61 (49,78) | < 0.001^**^ | |
| TMTB | 188.5 (144.0,256.8) | 160 (126,208) | < 0.001^**^ | |
| DSTF | 7 (6,8) | 8 (7,8) | < 0.001^**^ | |
| DSTB | 3 (3,4) | 4 (3,5) | < 0.001^**^ | |
| PMT | 11 (8,13) | 12 (10,14) | < 0.001^**^ | |
| SCWT-IT | 37 (28,51.5) | 36 (28,47) | 0.098 | |

Data shown as median (interquartile range) were compared using Mann –Whitney U test and data shown as *n* (%) were compared using the chi-square test or Fisher’s exact test

MCI, mild cognitive impairment; NC, normal cognition; BMI, body mass index; CVD, cerebrovascular diseases; MMSE, the Mini-Mental State Examination; AVLT-IR, Auditory Verbal Learning Test—immediate recall; AVLT-SR, Auditory Verbal Learning Test—short recall; AVLT-LR Auditory Verbal Learning Test—long recall; SDMT, Symbol Digit Modalities Test; LMT, Logical Memory Test; TMTA(B), Trail Making Test A(B); DSTF, digit span test forwards; DSTB, digit span test backwards; PMT, Picture Memories Test; SCWT-IT, Stroop Color-Word Test Interference Trial

^∗^ *P* < 0.05. ^∗∗^ *P* < 0.001

**Supplementary Table 2 Comparison of general characteristics between overall and subgroup**

| **Variables** | **Overall** | **Subgroup** | ***P* value** |
| --- | --- | --- | --- |
| **General characteristics** | | | |
| Age | 60(57,64) | 61(58,64) | 0.481 |
| Sex  Male, *n* (%)  Female, *n* (%) | 1158 (45.5%)  1388 (54.4%) | 34 (32.7%)  70 (67.3%) | 0.010^*^ |
| Education years | 9(9,12) | 9(9,12) | 0.231 |
| BMI (kg/m^2^) | 24.5(22.8,26.6) | 25.6(23.7,27.5) | 0.001^*^ |
| Smoking, *n* (%) | 642 (25.2%) | 29 (27.9%) | 0.115 |
| Drinking, *n* (%) | 1349 (56.0%) | 28 (26.9%) | < 0.001^**^ |
| Hypertension, *n* (%) | 914 (35.9%) | 37 (35.9%) | 1.000 |
| Hyperlipidemia, *n* (%) | 738 (29.0%) | 27 (26.2%) | 0.849 |
| Diabetes, *n* (%) | 459 (18.0%) | 15 (14.6%) | 0.564 |
| CVD, *n* (%) | 65 (2.6%) | 0 (0%) | 0.284 |
| **Nutrients and food intake** | | | |
| Cholesterol, mg | 275.3(156.1,340.0) | 274.5(173.0,345.0) | 0.867 |
| Vitamin B_12_, μg | 1.1(0.6,1.8) | 1.3(0.6,1.8) | 0.723 |
| Meat, g | 41.0(18.0,78.3) | 36.6(14.3,68.0) | 0.426 |
| Eggs and milk, g | 96.4(50.0,225.0) | 67.6(50.0,198.2) | 0.296 |
| **Cognitive function** | | | |
| MMSE | 28.0(27.0,29.0) | 28.0(27.0,29.0) | 0.781 |

Data shown as median (interquartile range) were compared using Mann–Whitney U test and data shown as n (%) were compared using the chi-square test or Fisher’s exact test between overall and subgroup study

BMI, body mass index; CVD, cerebrovascular diseases; MMSE, the Mini-Mental State Examination

^∗^ *P* < 0.05. ^∗∗^ *P* < 0.001

**Supplementary Table 3 Mediation effect of cholesterol oxygenated derivatives or tau phosphorylation on cholesterol or vitamin B_12_ and MMSE**

| **Mediator** | **ACME** | | **Prop. Mediated** | |
| --- | --- | --- | --- | --- |
|  | **Estimate(95%CI)** | ***P* value** | **Estimate(95%CI)** | ***P* value** |
| 27-OHC | 0.003(-0.102, 0.120) | 0.960 | 0.000(-0.694, 0.670) | 0.990 |
| 24S-OHC | -0.054(-0.289, 0.140) | 0.570 | 0.079(-1.420, 1.830) | 0.660 |
| t-tau | -0.054(-0.265, 0.120) | 0.530 | 0.078(-1.479,1.920) | 0.610 |
| p-tau | -0.017(-0.235, 0.180) | 0.880 | -0.029(-2.193, 2.590) | 0.860 |
| 27-OHC | 0.004(-0.092,0.110) | 0.970 | 0.002(-0.829, 0.960) | 0.970 |
| 24S-OHC | -0.045(-0.265, 0.100) | 0.590 | 0.036(-1.404, 2.640) | 0.730 |
| t-tau | -0.014(-0.215,0.200) | 0.860 | 0.039(-1.414,1.620) | 0.800 |
| p-tau | 1.89e-02(-1.61e-01, 0.200) | 0.820 | 7.55e-05(-1.50e+00, 0.200) | 0.990 |

Adjusted for age, sex, education, BMI, smoking, drinking, hypertension, hyperlipidemia, and diabetes using causal mediation analysis

MMSE, the Mini-Mental State Examination; t-tau, total tau; p-tau, phosphorylated tau; 27-OHC, 27-hydroxycholesterol; 24S-OHC, 24S-hydroxycholesterol; ACME, average causal mediation effect; CI, confidence interval

^∗^ *P* < 0.05. ^∗∗^ *P* < 0.001

**Supplementary Table 4 Log-binomial regression of meat or eggs/milk and MCI**

|  | **RR (95% CI)** | ***P* value** |
| --- | --- | --- |
| **Unadjusted** |  |  |
| Meat | 0.998(0.996,1.000) | 0.021^*^ |
| Eggs and milk | 0.946(0.820,1.093) | 0.454 |
| **Model 1** |  |  |
| Meat | 0.998(0.996,1.000) | 0.023^*^ |
| Eggs and milk | 0.942(0.816,1.088) | 0.417 |
| **Model 2** |  |  |
| Meat | 0.998(0.996,1.000) | 0.018^*^ |
| Eggs and milk | 0.946(0.821,1.090) | 0.444 |

Model 1 was adjusted for age, sex, education, BMI, smoking, drinking;

Model 2 was adjusted for Model 1 and hypertension, hyperlipidemia, diabetes and CVD

MCI, mild cognitive impairment; BMI, body mass index; CVD, cerebrovascular disease; RR, risk ratio; CI, confidence interval

^∗^ *P* < 0.05. ^∗∗^ *P* < 0.001

**Supplementary Table 5 The association between meat and multi-domain cognitive function**

| **Variables** | **β (95% CI)** | ***P*_ linear** | ***P*_ nonlinear** | ***P*_ overall** |
| --- | --- | --- | --- | --- |
| MMSE | 0.001(0.001,0.002) | 0.036^*^ | 0.092 | 0.232 |
| AVLT-IR | 0.001(-0.002,0.003) | 0.548 | 0.524 | 0.377 |
| AVLT-SR | 0.001(0.001,0.002) | 0.215 | 0.455 | 0.544 |
| AVLT-LR | 0.001(-0.001,0.001) | 0.978 | 0.470 | 0.315 |
| SDMT | 0.002(-0.003,0.008) | 0.414 | 0.847 | 0.729 |
| LMT | 0.001(-0.001,0.004) | 0.328 | 0.404 | 0.477 |
| TMTA | 0.004(-0.009,0.016) | 0.593 | 0.793 | 0.978 |
| TMTB | 0.018(-0.018,0.055) | 0.324 | 0.572 | 0.967 |
| DSTF | 0.000(0.000,0.001) | 0.247 | 0.683 | 0.629 |
| DSTB | -0.001(-0.001,0.000) | 0.081 | 0.341 | 0.281 |
| PMT | 0.001(-0.001,0.002) | 0.476 | 0.575 | 0.387 |
| SCWT-IT | 0.001(-0.009,0.012) | 0.808 | 0.974 | 0.899 |

Adjusted for age, sex, education, BMI, smoking, drinking, hypertension, hyperlipidemia, diabetes and CVD using multiple linear regression and RCS models

MMSE, the Mini-Mental State Examination; AVLT-IR, Auditory Verbal Learning Test—immediate recall; AVLT-SR, Auditory Verbal Learning Test—short recall; AVLT-LR Auditory Verbal Learning Test—long recall; SDMT, Symbol Digit Modalities Test; LMT, Logical Memory Test; TMTA(B), Trail Making Test A(B); DSTF, digit span test forwards; DSTB, digit span test backwards; PMT, Picture Memories Test; SCWT-IT, Stroop Color-Word Test Interference Trial; BMI, body mass index; CVD, cerebrovascular diseases; CI, confidence interval

^∗^ *P* < 0.05. ^∗∗^ *P* < 0.001
